# Expression of Drug Targets in Patients Treated with Sorafenib, Carboplatin and Paclitaxel

**DOI:** 10.1371/journal.pone.0069748

**Published:** 2013-08-06

**Authors:** Lucia B. Jilaveanu, Fengmin Zhao, Christopher R. Zito, John M. Kirkwood, Katherine L. Nathanson, Kurt D'Andrea, Melissa Wilson, David L. Rimm, Keith T. Flaherty, Sandra J. Lee, Harriet M. Kluger

**Affiliations:** 1 Yale Cancer Center, Yale University School of Medicine, New Haven, Connecticut, United States of America; 2 Department of Biostatistics and Computational Biology, Dana-Farber Cancer Institute, Boston, Massachusetts, United States of America; 3 Department of Biology, School of Health and Natural Sciences, University of Saint Joseph, West Hartford, Connecticut, United States of America; 4 Pittsburgh Hillman Cancer Center, University of Pittsburgh, Pittsburgh, Pennsylvania, United States of America; 5 Abramson Cancer Center, University of Pennsylvania, Philadelphia, Pennsylvania, United States of America; 6 Department of Pathology, Yale University School of Medicine, New Haven, Connecticut, United States of America; 7 Department of Medicine, Massachusetts General Hospital Cancer Center, Boston, Massachusetts, United States of America; The Moffitt Cancer Center & Research Institute, United States of America

## Abstract

**Introduction:**

Sorafenib, a multitarget kinase inhibitor, targets members of the mitogen-activated protein kinase (MAPK) pathway and VEGFR kinases. Here we assessed the association between expression of sorafenib targets and biomarkers of taxane sensitivity and response to therapy in pre-treatment tumors from patients enrolled in ECOG 2603, a phase III comparing sorafenib, carboplatin and paclitaxel (SCP) to carboplatin, paclitaxel and placebo (CP).

**Methods:**

Using a method of automated quantitative analysis (AQUA) of in situ protein expression, we quantified expression of VEGF-R2, VEGF-R1, VEGF-R3, FGF-R1, PDGF-Rβ, c-Kit, B-Raf, C-Raf, MEK1, ERK1/2, STMN1, MAP2, EB1 and Bcl-2 in pretreatment specimens from 263 patients.

**Results:**

An association was found between high FGF-R1 and VEGF-R1 and increased progression-free survival (PFS) and overall survival (OS) in our combined cohort (SCP and CP arms). Expression of FGF-R1 and VEGF-R1 was higher in patients who responded to therapy ((CR+PR) vs. (SD+PD+ un-evaluable)).

**Conclusions:**

In light of the absence of treatment effect associated with sorafenib, the association found between FGF-R1 and VEGF-R1 expression and OS, PFS and response might reflect a predictive biomarker signature for carboplatin/paclitaxel-based therapy. Seeing that carboplatin and pacitaxel are now widely used for this disease, corroboration in another cohort might enable us to improve the therapeutic ratio of this regimen.

## Introduction

The incidence of melanoma is rising faster than that of any other malignancy; the incidence of metastatic disease and death are rising as well [1]. Treatment of advanced disease has been a challenge and so far has shown only limited efficacy. Until 2010, no therapies studied in randomized trials had an impact on OS, including chemotherapy, biological therapies and combinations of both. [2]. Recently, a monoclonal antibody that inhibits CTLA-4, ipilimumab (Bristol Myers Squibb and Medarex Incorporated), showed durable objective responses and improved median survival in some patients when compared with a peptide vaccine or DTIC [3], [4]. The second major recent advance was in selective targeting of mutated B-Raf. The MAPK pathway is activated in the majority of human melanomas and plays a critical role in regulating the proliferation, invasion and survival of melanoma cells; approximately half of the melanomas harbor activating mutations in B-Raf and 15–20% have mutations in NRAS [5]. Thus, drugs that target the MAPK pathway have been the focus of intense clinical research. One selective inhibitor of mutant B-Raf, PLX4032 (RG7204/RO5185426/Vemurafenib, Genentech) has been recently approved for treatment of metastatic melanoma after showing remarkable clinical activity in patients with mutated B-Raf when compared to dacarbazine [6]. Other MAPK pathway inhibitors, such as GSK2118436 (Dabrafenib, GlaxoSmithKline), also a selective inhibitor of mutant B-Raf, and GSK1120212 (Trametinib, GlaxoSmithKline), a potent MEK inhibitor, have been investigated in advanced clinical trials for patients with melanoma harboring B-Raf mutations and showed to improve survival when compared to chemotherapy [7], [8].

Sorafenib (BAY 43-9006, Nexavar, Bayer Pharmaceuticals Corporation & Onyx Pharmaceuticals) is an orally active, unselective, multikinase agent that inhibits C-Raf and B-Raf (mutant and wild type) along with a number of other cellular proteins involved in tumor neovascularization and tumor cell proliferation and survival, including VEGFR-2, VEGFR-3, Flt3, FGFR1, PDGFR-β, c-Kit and p38α [9]. Sorafenib is FDA approved for treatment of advanced renal cell carcinoma (RCC) and hepatocellular carcinoma (HCC) [10], [11]. In pre-clinical melanoma models (cell lines and tumor xenografts) sorafenib slowed cellular proliferation and tumor growth through inhibitory effects on the MAPK pathway [9], [12], [13], [14], [15]. Sorafenib was therefore felt to be a reasonable drug to study in melanoma. In clinical trials, single agent sorafenib had little activity in melanoma patients, with response rates of less than 10% in two early phase studies [16], [17]. However, in a phase I multi-tumor study in which sorafenib was combined with carboplatin and paclitaxel (SCP), a number of responses were seen in melanoma patients, leading to an expanded phase I/II trial of SCP in melanoma [18]. This study demonstrated an overall response rate of 26% in melanoma patients and a median PFS of 307 days, a result that required validation in a phase III trial. Two such trials were conducted; a second line therapy study in which SCP was compared to carboplatin, paclitaxel and placebo (CP) and a cooperative group study for patients who were chemotherapy-naive led by the Eastern Cooperative Oncology Group (ECOG), called E2603 [19], [20], [21]. Both of these trials failed to demonstrate a benefit in OS or PFS for SCP versus CP plus placebo.

Retrospective analysis of B-Raf mutational status in patients treated on the phase I/II trial showed no difference in activity of SCP in patients with B-Raf mutated tumors compared to B-Raf wild-type (WT) tumors [18]. To identify potential predictors of response to SCP, we previously quantitatively assessed the expression of targets of sorafenib in pretreatment tumors from 44 patients enrolled in the phase I/II trial of this multidrug regimen. In this small cohort we found that high levels of VEGF-R2 and low ERK1/2 levels were associated with a greater likelihood of response in patients treated with SCP [22]. that none of the patients in this cohort were treated with CP alone, it was unclear whether this association was related to chemotherapy sensitivity or sensitivity to the combination regimen.

The purpose of this current study was to assess expression of direct and indirect sorafenib targets in pretreatment specimens from patients enrolled in E2603. We also assessed expression levels of select molecules that have previously been shown to be associated with sensitivity to taxane drugs (Stathmin (STMN1), Microtubule-associated protein 2 (MAP2), End-binding protein 1(EB1) and Bcl-2). Specimens were available for 263 patients. We evaluated the association between marker expression and efficacy outcomes (objective response rate (ORR), OS and PFS) in the 263 patients. Marker expression was assessed using an objective, method of automated, quantitative analysis, AQUA (Automated Quantitative Analysis). This method has been validated for epithelial cancers and melanoma and proven to be superior to pathologist-based scoring of 3,3'-diaminobenzidine stain in that it is more precise, highly reproducible and quantitative [23], [24].

## Materials and Methods

### Patients and study design

E2603 enrolled 823 patients. Specimen collection was conducted as part of the trial and analyzed with institutional review board approval. All patients had pathologically confirmed advanced unresectable or metastatic melanoma without brain involvement and had not previously been treated with chemotherapy or MAPK pathway targeted drugs. The study design, including the patient details, has been described previously [21]. Patients had measureable disease by RECIST, were at least 18 years of age, had an ECOG performance status of 0 or 1, and satisfactory baseline organ function. Patients were enrolled without determination of B-Raf or N-Ras mutational status.

### Treatment and assessment of response

Patients were randomized in a double-blinded fashion to receive either SCP or CP. On day 1 of each 21 day cycle (cycles 1 through 4), paclitaxel was administered at 225 mg/m2 IV over 3 hours followed by carboplatin AUC 6 IV over 30 minutes. Sorafenib (400 mg) or placebo was given by mouth twice daily days 2 through 19 of each 21 day cycle. On day 1 of each 21 day cycle (cycles 5 through 10), paclitaxel was administered at 175 mg/m2 IV over 3 hours followed by carboplatin AUC 5 IV over 30 minutes. Sorafenib (400 mg) or placebo was given by mouth twice daily on days 2 through 19 of each 21 day cycle. Upon completion of 10 cycles of chemotherapy, Sorafenib (400 mg BID) or placebo was administered daily until disease progression or intolerable toxicity.

Tumor response was assessed every 2 cycles during cycle 1 to 10 and then every 3 cycles. Response was defined by RECIST.

### Tissue microarray (TMA) construction

Pre-treatment formalin-fixed paraffin embedded tumor biopsies were obtained from patients as part of the clinical trial. A pathologist (DLR) examined each case and selected a representative region of invasive tumor to be included in the array. Adequate tissue was available on 263 patients. Two cores from each block were taken to construct the TMAs as previously described [25], [26]. Cores measuring 0.6 mm in diameter, were spaced 0.8 mm apart. The tissue microarrays were cut into 5-µm sections and placed on glass slides using an adhesive tape-transfer system with UV cross-linking. To account for experimental variation and for normalization across array blocks, pellets of melanoma cell lines were embedded in all array blocks, as described previously [27].

### Immunofluorescence

One set of two slides (each containing a core from different areas of tumor for the same patient) was stained for VEGF-R2, VEGF-R1, VEGF-R3, FGF-R1, PDGF-Rβ, c-Kit, B-Raf, C-Raf, MEK1, ERK1/2, STMN1, MAP2, EB1 and Bcl-2. Staining was carried out for AQUA as described [25], [28]. A list of antibodies and manufacturers is supplied in Table S1 in File S1. Briefly, slides were deparaffinized in xylene followed by two rinses in 100% ethanol. Antigen retrieval was performed by boiling slides in a pressure cooker filled with 6.5 mM sodium citrate (pH 6.0). Slides were incubated in a mixture of methanol and 2.5% hydrogen peroxide for thirty minutes at room temperature to block the endogenous peroxidase activity. To block non-specific staining, slides were then incubated at room temperature for 30 minutes in 0.3% bovine serum albumin/1X Tris-buffered saline. Slides were incubated with the primary antibody diluted in Tris-buffered saline containing 0.3% bovine serum albumin at 4°C overnight. Slides were then washed three times in 1X Tris-buffered saline/0.05% Tween-20. Either goat anti-mouse (for rabbit primary antibodies) or goat anti-rabbit (for mouse primary antibodies) horseradish peroxidase-decorated polymer backbone (Envision, Dako) was utilized to visualize the target protein. To create a tumor mask, slides were simultaneously incubated with either mouse or rabbit anti-S100 and anti-HMB45, at 1∶100. For visualization of tumor mask staining a goat anti-mouse or anti-rabbit IgG conjugated to Alexa 546 (Molecular Probes, Inc.) at 1∶200 was utilized. Primary antibody staining was visualized with Cy5-tyramide (NED Life Science Products). Coverslips were mounted with ProLong Gold antifade reagent with 4′,6-diamidino-2-phenylindole (DAPI) (Invitrogen).

### Automated image acquisition and analysis

Images were analyzed using algorithms that have been extensively described [23]. Briefly, monochromatic, high-resolution (1280×1024 pixel) images were obtained of each histospot. Tumor was distinguished from stromal elements by S-100 and HMB45 signal. Coalescence of S-100 and HMB45 at the cell surface was used to localize cell membrane/cytoplasm compartment within the tumor mask, and DAPI was used to identify the nuclear compartment within the tumor mask. Targets were visualized with Cy5. Images were obtained for each histospot using the 10X objective of an Olympus AX-51 epifluorescence microscope (Olympus, Melville, NY) with an automated microscope stage and digital image acquisition driven by custom program and macrobased interfaces with IPLabs software (Scanalytics, Inc., Fairfax, VA). The signal intensity for all targets was scored on a scale of 0–255 (the AQUA score). Histospots with limited amount of tumor tissue (<3%) were excluded from the analysis.

### Data analysis

Expression of markers was analyzed using either continuous AQUA scores or variables dichotomized at the median, reflecting the use of routine statistical divisions in the absence of an underlying justification for division of expression. The Pearson correlation test was used to compare AQUA scores for markers from matching spots from the two arrays. The association between dichotomized markers and objective response status (complete response (CR)+ partial response (PR) vs. stable disease (SD)+ progressive disease (PD)+ un-evaluable) and clinical/pathological parameters (AJCC stage, ECOG performance status, LHD level, prior therapy) was examined by the Fisher's exact test. The association between continuous AQUA scores and objective response and clinical/pathological parameters was assessed by two-sample t tests (objective response, ECOG PS and LDH level) and analysis of variance (AJCC stage and prior treatment). Kaplan-Meier methods were used to estimate OS and PFS distribution, and log-rank test was used to test significance in distribution difference between patients with high or low scores for each marker (dichotomized at median) or between treatment arms. OS was defined as time from randomization to death from any cause, censoring patients that were alive at the last follow-up date. PFS was defined as time from randomization to disease progression or death (whichever occurred first), censoring cases without progression at the date of last disease assessment. Univariate Cox proportional hazards methods were used to estimate the unadjusted hazards ratios for OS and PFS for all markers using continuous AQUA scores and dichotomized variables. For markers which were significant on univariate analysis for OS or PFS, multivariable Cox models were used to estimate the adjusted hazard ratios (dichotomized at median), adjusting for AJCC stage, ECOG PS, prior treatment, number of disease sites involved, and LDH.

All p-values were two sided and confidence intervals were at the 95% level. No adjustment was made for multiple comparisons. SAS version 9.2 software was used for all analysis (SAS, Cary, NC).

### Ethics statement

This work was approved by the Yale University Institutional Review Board. In addition, approval was obtained through the Eastern Cooperative Oncology Group (ECOG) for use of and clinical data from patients enrolled in E2603 clinical trial. Written informed consent was obtained from all patients on this trial.

## Results

The demographics for the 263 patients are summarized in Table S2 in File S1. There was no statistically significant difference regarding known prognostic factors between the 263 patients and the 560 patients without the biomarker data (excluded from the current report). The two groups of patients also had similar outcomes (Table S3 in File S1).

To assess the association between marker expression and clinical response to therapy, we stained our TMAs with antibodies to VEGF-R2, VEGF-R1, VEGF-R3, FGF-R1, PDGF-Rβ, c-Kit, B-Raf, C-Raf, MEK1, ERK1/2, STMN1, MAP2, EB1 and Bcl-2. Antibodies were previously validated by Western blots of lysates obtained from a panel of melanoma cell lines to verify specificity [22] (antibody specifications are provided in Table S1 in File S1). We note that a paper was recently published suggesting the VEGF-R2 antibody by Santa Cruz Technologies used in our previous paper was not specific, and that another antibody made by Cell Signaling Technologies was superior. We therefore purchased the Cell Signaling antibody and found the staining with this antibody to be non-specific and irreproducible, while the Western blot using the Santa Cruz antibody yielded a single band of the appropriate size and staining was both reproducible and specific. We therefore used the Santa Cruz antibody for these studies. With the exception of ERK1/2 which showed both nuclear and cytoplasmic staining, all markers were membranous/cytoplasmic and this compartment was therefore analyzed. Staining patterns within the tumor mask within a histospot were fairly homogenous for all markers analyzed. The range of AQUA scores and mean values for each marker are provided in Table S4 in File S1. We used the Pearson correlation test to compare scores from matching spots from the two arrays. For all markers, expression on two matching specimens from the different arrays was found to be highly correlated (R values >0.5 and P<0.0001 for all). For patients with multiple available blocks, the different blocks were cored and AQUA scores were averaged to give one AQUA score per patient. For each of the markers, the AQUA scores from both sets of slides were combined to give a single dataset. Tumor spots were deemed un-interpretable if they had insufficient tumor cells, loss of tissue in the spot, or an abundance of necrotic tissue. For patients who had two interpretable histospots, a composite score was formed by calculating the mean of the two scores. For patients with only one interpretable core, the single score was used for analysis. Tumor specimens were available for a total of 335 patients in E2603, of which 72 had tumors that were too small or too necrotic for use in the biomarker studies and therefore 263 patients with interpretable histospots were included in the current analysis. The sample size for the combined dataset for each marker is provided in Table S4 in File S1.

We assessed associations between sorafenib targets by Pearson correlation, as demonstrated in Table S5 in File S1. Expression of most of the markers was found to be correlated, as expected, given that many of them are in the MAPK pathway.

**Figure 1:**
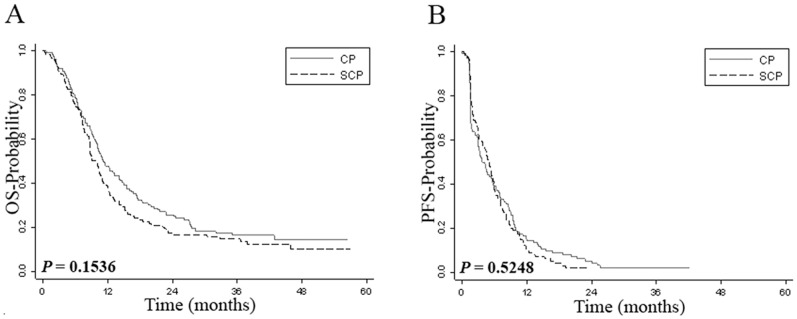
Kaplan-Meier survival analysis by treatment arm. Panel A shows Kaplan-Meier estimates of overall survival while panel B shows Kaplan-Meier estimates of progression-free survival for patients treated with sorafenib, carboplatin and paclitaxel (SCP) or carboplatin and paclitaxel (CP).

Among the patients with available tissue, the ORR, which included CR and PR, was not statistically different between the SCP (123 patients) and CP (140 patients) arms (17.9% vs. 16.4%, *P* = 0.87). Of these, 24 patients (11/123 on SCP arm and 13/140 on CP arm) had an un-evaluable tumor response. We also found no difference in OS and PFS between the two arms (Figure 1). These results indicate that activity of SCP and CP in the group of patients with available tissue was similar to that of the entire patient cohort.

In our previous studies on a subset of specimens of patients treated with SCP in the phase I/II trial, we found that low ERK1/2 levels were associated with improved ORR, OS and PFS compared to high ERK1/2 levels, whereas high VEGF-R2 levels were associated with a higher ORR to SCP. the E2603 treated patients, low ERK1/2 or/and high VEGF-R2 were not significantly associated with better clinical outcomes or increased ORR, PFS and OS in the SCP arm. To assess the combined effect of high VEGF-R2 and low ERK1/2 on response, we next generated a composite VEGF-R2/ERK1/2 score by dichotomizing AQUA scores by the associated median score for each marker, as we did previously [22]. We then defined three groups of patients: one group had low VEGF-R2 and high ERK1/2 expression, the second group had either low VEGF-R2 and low ERK1/2 or high VEGF-R2 and high ERK1/2, and the third group had high VEGF-R2 and low ERK1/2. We found no association between this composite VEGF-R2/ERK1/2 score and ORR (*P* = 0.15 for all patients; *P* = 0.60 for SCP arm, *P* = 0.22 for CP arm). The phase I/II trial with SCP included more patients with a worse prognosis and high LDH. We therefore studied the association between the composite VEGF-R2/ERK1/2 score and tumor response (ORR) in patients with an elevated LDH at registration (81 patients), and there was no significant association (*P* = 0.30 for all patients, *P* = 0.29 for SCP arm, *P* = 0.23 for CP arm).

Since expression of ERK1/2 and/or VEGF-R2 was not associated with response or survival in the sorafenib arm and since distributions of OS and PFS between the two arms were similar, we combined the two patient sub-sets and tested associations between marker expression and response or survival in the whole cohort of 263 patients. We note that the patients in the two treatment arms were well balanced in regard to response, survival and other clinical variables, and no significant differences were noted between the subsets on the two arms (Tables S2 and S3 in File S1). To assess the association between marker expression and ORR we performed two-sample t test using the continuous AQUA scores comparing responders (CR+PR) with non-responders (SD+PD+ un-evaluable). The expression of VEGF-R1 was significantly higher in patients who responded to therapy (CR+PR) than in non-responding patients (*P* = 0.02) (Table 1). A trend toward significance was seen for FGF-R1 (*P* = 0.08). Although expression of ERK1/2 was slightly lower in patients who achieved a clinical response, this association did not reach statistical significance (*P* = 0.27). There was no statistically significant association between tumor expression of other markers and response to therapy (Table 1). When dichotomized marker score was used, VEGF-R1 expression was significantly correlated with response (69.4% responders had high expression of VEGF-R1, and the proportion was 46.0% in non-responders, *P* = 0.011). For all other markers, there was no significant association.

**Table 1:** Average level of expression for each biomarker (continuous AQUA scores) by response status.

	Non-Responders		Responders		
Marker	Mean	SD	Mean	SD	P value
B-Raf	45.5	16.8	46.7	14.4	0.71
c-Kit	38.8	13.1	38.6	10.5	0.93
C-Raf	25.9	11.5	23.8	9.3	0.29
**FGF-R1**	**68.1**	**16.5**	**73.0**	**16.3**	**0.08**
**VEGF-R1**	**33.2**	**14.3**	**39.6**	**17.7**	**0.02**
VEGF-R3	51.6	18.1	55.0	19.4	0.28
MEK1	40.2	16.6	40.7	16.2	0.86
PDGF-Rβ	42.5	15.1	42.7	11.7	0.92
STMN1	55.6	19.8	57.0	16.3	0.72
MAP2	24.8	19.4	29.1	24.2	0.30
EB1	37.4	19.4	37.5	19.5	0.99
Bcl-2	27.2	18.9	22.3	13.2	0.13
ERK1/2	36.4	18.5	32.8	13.1	0.27
VEGF-R2	30.5	10.5	30.1	10.7	0.84

Note: P values were generated from two- independent sample t tests.

To assess the association between marker expression and clinical/pathological parameters at registration, we used two-sample t tests for ECOG PS (0 vs. 1–2) and LDH level (normal vs. elevated) and analysis of variance for AJCC stage (Stage III, M1a/1b, M1c) and prior treatment (none, IFN/IL-2/GM-CSF, one investigational therapy). We found that high VEGF-R2, high EB1 and high PDGF-Rβ expression were significantly correlated with more advanced AJCC stage (*P* = 0.0099, *P* = 0.0087, *P* = 0.032, respectively). VEGF-R1 expression was significantly associated with no prior treatment (*P* = 0.027) (Table 2). For other markers, there was no significant association. When the Fisher exact test was used to test the association between dichotomized marker scores and clinical/pathological parameters, high ERK1/2 and high VEGF-R2 expression were significantly correlated with more advanced AJCC stage (*P* = 0.043 for ERK, P = 0.004 for VEGFR2) (Table 3). For all other markers, there was no significant association between expression and clinical variables.

**Table 2:** Average level of expression for each biomarker (continuous AQUA scores) by clinical parameters.

	AJCC			Prior Therapy			PS		LDH	
Marker	Stage III	M1a/1b	M1c	None	IFN/IL2/GM-CSF	One	1	0	Normal	Elevated
B-Raf	42.3	43.6	47.9	47.1	43.9	47.9	45.8	45.6	46.2	45.5
c-Kit	37.1	37.0	40.2	39.8	37.5	36.8	39.3	37.6	38.7	39.0
C-Raf	26.6	24.4	26.2	27.1	23.7	27.0	26.1	24.7	25.4	26.5
FGF-R1	64.6	68.5	70	70.0	67.8	64.8	69.1	68.6	68.7	69.3
**VEGF-R1**	35.3	33.7	34.3	**36.2**	**32.2**	**18.7**	34.1	34.4	33.3	35.8
VEGF-R3	52.0	52.9	51.7	54.2	49.2	53.3	52.0	52.3	52.5	52.3
MEK1	39.5	39.7	40.7	42.1	38.0	32.2	41.1	38.5	41.7	38.5
**PDGF-Rβ**	**38.5**	**40.4**	**44.9**	43.2	41.5	44.8	41.8	43.9	41.6	44.1
STMN1	50.8	54.5	57.7	57.1	54.5	50.2	55.6	56.1	55.7	56.6
MAP2	23.7	27.4	24.4	25.5	25.3	25.8	25.0	26.1	26.7	22.6
**EB1**	**29.6**	**34.9**	**40.7**	39.0	35.8	29.4	38.4	35.6	38.1	37.1
Bcl-2	26.4	27.2	25.9	28.6	23.5	33.0	26.3	26.7	26.3	27.3
ERK1/2	31.0	36.6	36.4	36.4	35.2	31.5	37.3	33.0	37.4	34.3
**VEGF-R2**	**27.7**	**28.4**	**32.4**	31.5	29.0	32.0	30.5	30.4	30.5	30.9

Note: significant correlations (*P*<0.05 for two-independent sample t test or analysis of variance test) are marked in bold.

**Table 3:** Percentage of tumors with high expression for each biomarker by clinical parameters (AQUA scores dichotomized at the median).

	AJCC			Prior Therapy			PS		LDH	
Marker	Stage III	M1a/1b	M1c	None	IFN/IL-2/GM-CSF	One	1	0	Normal	Elevated
B-Raf	42.3	45.5	54.8	52.5	45.8	75.0	51.1	48.0	51.9	48.8
c-Kit	48.2	45.1	53.5	55.0	44.1	40.0	50.3	49.4	49.6	51.7
C-Raf	69.2	42.7	50.4	53.9	45.1	40.0	52.4	45.4	48.2	54.4
FGF-R1	30.8	48.3	54.6	50.4	50.5	33.3	46.6	55.9	48.0	53.1
VEGF-R1	61.5	46.3	49.6	50.4	50.5	0.0	51.4	46.8	49.6	50.6
VEGF-R3	46.2	51.8	49.2	56.4	41.0	50.0	47.4	54.1	51.1	50.0
MEK1	44.0	52.0	49.6	53.6	45.7	25.0	51.8	46.3	53.9	45.4
PDGF-Rβ	40.7	41.8	57.1	54.8	43.9	33.3	47.3	54.4	47.7	53.4
STMN1	50.0	42.0	54.7	51.4	48.9	25.0	49.2	50.7	51.7	49.3
MAP2	63.6	53.4	44.3	48.1	51.6	50.0	50.0	49.3	50.4	46.6
EB1	37.0	46.3	54.8	55.1	43.6	40.0	51.0	47.6	51.8	48.9
Bcl-2	44.0	46.9	52.8	52.8	44.7	80.0	50.3	48.8	50.7	50.6
**ERK1/2**	**26.9**	**52.6**	**53.4**	50.4	50.0	33.3	51.8	46.8	51.2	51.2
**VEGF-R2**	**38.5**	**38.6**	**59.8**	50.8	49.0	40.0	49.0	51.1	49.3	50.5

Note: significant correlations (*P*<0.05 for Fisher exact test) are marked in bold.

We next assessed the association between expression of each marker and OS by Cox proportional hazards models. When dichotomized AQUA scores were used, high FGF-R1 was associated with lower hazards for OS (HR = 0.75, 95% CI: 0.57–0.98, *P* = 0.038), and VEGF-R1 and c-Kit had a trend toward significance (HR = 0.75, 95% CI: 0.56–1.00, *P* = 0.052 for VEFG-R1, HR = 0.77, 95%CI: 0.59–1.02, *P* = 0.073 for c-Kit) (Table 4). Kaplan Meier curves are provided in Figure S1 in File S1. No significant association was found for other markers. When continuous AQUA scores were used in the univariate Cox regression analysis, FGF-R1 remained significantly associated with survival (*P* = 0.004), and VEGF-R1 and c-Kit became significant as well (*P* = 0.012 and *P* = 0.041, respectively) (Table 4). Using multivariable Cox analysis, we found that high FGF-R1, high VEGF-R1, and high c-Kit expression were also independent predictors of better survival (*P* = 0.0019 for FGF-R1, *P* = 0.0058 for VEGF-R1, and *P* = 0.0205 for c-Kit) (Tables S6, S7 and S8 in File S1). The only other variables associated with OS by multivariable analysis were “prior treatment” status and LDH for FGF-R1 (*P* = 0.0499 and *P* = 0.0003, respectively), “site number” status and LDH for VEGF-R1 (*P* = 0.0388 and *P* = 0.0031, respectively) and for c-Kit (*P* = 0.029 and *P* = 0.001, respectively). All other variables included in the model were not associated with OS.

**Table 4:** Univariate Cox regression analysis of dichotomized and continuous AQUA scores for OS.

	Dichotomized AQUA Scores			Continuous AQUA Scores*		
Marker	Hazard Ratio	95% CI	P value	Hazard Ratio	95% CI	P value
B-Raf	0.89	0.66–1.19	0.4175	0.99	0.90–1.09	0.8185
**c-Kit**	0.77	0.59–1.02	0.0728	**0.89**	**0.80**–**1.00**	**0.0410**
C-Raf	0.93	0.70–1.23	0.5961	0.91	0.80–1.03	0.1470
**FGF-R1**	**0.75**	**0.57**–**0.98**	**0.0381**	**0.86**	**0.79**–**0.93**	**0.0004**
**VEGF-R1**	**0.75**	**0.56**–**1.00**	**0.0517**	**0.88**	**0.80**–**0.97**	**0.0120**
VEGF-R3	1.03	0.78–1.36	0.8274	0.98	0.91–1.06	0.6869
MEK1	0.94	0.71–1.26	0.6883	0.96	0.87–1.05	0.3228
PDGF-Rβ	0.81	0.61–1.07	0.1421	0.92	0.83–1.02	0.1079
STMN1	1.09	0.80–1.48	0.5927	1.01	0.93–1.09	0.8337
MAP2	0.83	0.61–1.13	0.2359	0.95	0.88–1.03	0.1956
EB1	0.83	0.63–1.10	0.2028	0.97	0.90–1.05	0.4323
Bcl-2	1.05	0.80–1.39	0.7192	1.00	0.93–1.08	0.9552
ERK1/2	0.96	0.71–1.28	0.7564	0.95	0.88–1.03	0.2056
VEGF-R2	1.06	0.80–1.39	0.7012	0.95	0.83–1.08	0.4140

*Hazard ratios were calculated per 10-point change in AQUA scores.

Similar analysis was also conducted for PFS. When dichotomized AQUA scores were used, univariate Cox regression showed that high FGF-R1 and high EB1 were associated with lower hazards (HR = 0.76, *P* = 0.0306 for FGF-R1; HR = 0.77, *P* = 0.05 for EB1), and MAP2 had a trend toward significance (HR = 0.76, *P* = 0.0668) (Table 5). Kaplan Meier curves are provided in Figure S2 in File S1. No significant association was found for other markers. When continuous AQUA scores were used in the univariate Cox regression analysis for PFS, FGF-R1 remained significant (*P* = 0.0174); MEK1 and MAP2 became significant as well (*P* = 0.041 and *P* = 0.0294, respectively) (Table 5). VEGF-R2 had a trend toward significance (*P* = 0.072). Multivariable Cox analysis showed that FGF-R1 was also an independent predictor of increased PFS (*P* = 0.016) (Table S9 in File S1). No other variables were associated with PFS on multivariable analysis.

**Table 5:** Univariate Cox regression analysis of dichotomized and continuous AQUA scores for PFS.

	Dichotomized AQUA Scores			Continuous AQUA Scores*		
Marker	Hazard Ratio	95% CI	P value	Hazard Ratio	95% CI	P value
B-Raf	0.87	0.66–1.15	0.3399	0.95	0.87–1.04	0.2837
c-Kit	0.89	0.68–1.15	0.3666	0.93	0.83–1.03	0.1754
C-Raf	0.82	0.63–1.07	0.1482	0.97	0.85–1.10	0.6354
**FGF-R1**	**0.76**	**0.59**–**0.97**	**0.0306**	**0.90**	**0.83**–**0.98**	**0.0174**
VEGF-R1	0.83	0.63–1.09	0.1775	0.93	0.85–1.02	0.1217
VEGF-R3	0.87	0.67–1.14	0.3126	0.97	0.90–1.04	0.3827
**MEK1**	0.84	0.64–1.10	0.2127	**0.91**	**0.82**–**1.00**	**0.0410**
PDGF-Rβ	0.90	0.69–1.18	0.4410	0.95	0.86–1.05	0.2897
STMN1	0.95	0.71–1.26	0.7075	0.95	0.88–1.03	0.2393
**MAP2**	0.76	0.57–1.02	0.0668	**0.92**	**0.86**–**0.99**	**0.0294**
**EB1**	**0.77**	**0.59**–**1.00**	**0.0500**	0.97	0.90–1.05	0.4902
Bcl-2	1.02	0.79–1.33	0.8687	1.00	0.93–1.07	0.9231
ERK1/2	1.13	0.86–1.49	0.3790	1.00	0.92–1.08	0.9709
VEGF-R2	0.99	0.77–1.29	0.9569	0.89	0.79–1.01	0.0722

*Hazard ratios were per 10-point change in AQUA scores.

B-Raf and N-Ras mutational status was available for 111 of these patients, of whom 48 had B-Raf mutations and 26 had N-Ras mutations. The sample size was small and the HRs for FGF-R1, VEGF-R1 and c-Kit for survival and response were similar to those shown for the entire cohort of patients (data not shown).

## Discussion

Although the combination of sorafenib with carboplatin and paclitaxel showed promising results in the phase I/II trial, two subsequent double-blinded, randomized phase III studies did not confirm the initial promise of this regimen, although a trend towards a difference in OS and PFS between the two arms was seen in patients with an elevated LDH (P = 0.094 and P = 0.078, respectively) [18], [19], [20], [21]. In this retrospective biomarker study we sought to measure levels of sorafenib targets as well as select taxane targets, with the hope of further identifying a subset of patients that might benefit from SCP or CP.

We assessed associations between quantitative marker expression and response to therapy, PFS and OS, as well as with other commonly utilized clinical and pathologic variables. To the best of our knowledge, this is the largest prospectively collected melanoma cohort from a multicenter trial that has studied marker expression and its association with response to therapy, using continuous output scores, rather than arbitrary pathologist-based divisions of scores into high/low or strong/weak.

Our initial intention was to validate our previous observations of associations between low ERK1/2 and high VEGF-R2 and response to therapy seen in a subset of 44 patients treated in the phase I/II trial with SCP. In this larger patient cohort we did not confirm our previous findings. There are a number of possible explanations for this discrepancy. One possibility lies in the differences in trial design. As opposed to the phase I/II study which included a poorer prognosis group of patients (65% had elevated LDH levels and patients could have had progression of disease on up to five prior therapies), the phase III trial included chemotherapy-naive patients, of whom less than half had received prior immunotherapy, and 39% had elevated LDH levels. It is possible that high ERK1/2 is a measure of disease aggressiveness rather than response to therapy, and therefore more likely to have predictive value in a more advanced disease population. This hypothesis is supported by our previous studies on a historical cohort of melanoma patients treated at Yale University, in which we showed that high ERK1/2 is of prognostic value [22]. studies of VEGF-R2 expression in this cohort showed no association with survival, and in the phase I/II trial this marker was similarly not associated with PFS or OS [22], [29]. The other plausible explanation for the lack of consistency with our previous findings is the small size of the cohort in the early phase trials. The results (high response rate and higher PFS than expected) were not validated in the larger cohort used in this study, as is often the case when early phase trials show promising results that are not validated in larger randomized trials.

In the whole cohort of patients we found a statistically significant association between high FGF-R1 and VEGF-R1 and increased PFS and OS. Furthermore we found that expression of FGF-R1 and VEGF-R1 was significantly higher in patients who responded to therapy (CR+PR) than in non-responders (patients with SD+PD+ un-evaluable). The fact that patients with high FGF-R1 and VEGF-R1 were more likely to respond, is possibly due to inhibition of cellular proliferation driven by both autocrine loops, FGF2/FGF-R1 and VEGF/VEGF-R1. A number of studies including ours have implicated high expression of both FGF-R1 and VEGF-R1 in melanoma development and progression [29], [30]. In other diseases over-expression of FGF2, the ligand for FGF-R1, was shown to enhance apoptosis in MCF7 breast tumor cells exposed to chemotherapy such as cisplatin or 5-fluorouracil and to sensitize NIH 3T3 cells to apoptosis induced by cisplatin [31], [32], but this has not yet been proven to be the case for metastatic melanoma.

Approximately 50% of metastatic melanomas harbor activating B-Raf mutations, and 15–20% harbor activating N-Ras mutations [5]. In this phase III trial, B-Raf mutational status was available for 111 patients who also had specimens for AQUA analysis (48 had B-Raf mutations while 26 had N-Ras). Analysis of the association between marker expression and OS or PFS in the context of B-Raf or N-Ras mutational status yielded similar results to the ones obtained in the whole cohort of patients. *In vitro* data showed that sorafenib inhibits proliferation through MAPK pathway inhibition and angiogenesis [33] and approximately 75% of patients have constitutive MAPK pathway activation. The lack of efficacy of SCP in this phase III clinical study is likely due to the fact that sorafenib is not a highly selective or a potent B-Raf inhibitor; multiple sorafenib targets have been identified and many probably remain unknown. In fact, *in vitro* enzyme inhibition analyses show that the IC_50_ for mutated and wild-type B-Raf is 38 and 22 nmol/L respectively, while for VEGF-R2 it is 90 nmol/L [9]. In recent years, other more selective and more effective MAPK pathway inhibitors have emerged. Examples include PLX4032 (RG7204/RO5185426/Vemurafenib, Genentech), a selective inhibitor of mutant B-Raf, which was recently approved for treatment of metastatic melanoma, GSK2118436 (Dabrafenib, GlaxoSmithKline), a selective inhibitor of mutant B-Raf, and GSK1120212 (Trametinib, GlaxoSmithKline), a MEK inhibitor, which showed high response rates in patients with activating B-Raf mutations [7], [8]. Combinations of more selective MAPK pathway inhibitors and chemotherapy or ipilimumab warrant further evaluation. Another possible avenue worth investigating is the combination of sorafenib and drugs that potentiate sorafenib activity. *In vitro* studies have shown that certain inhibitors such as the farnesyl transferase inhibitor, ionafarnib, or statins, such as fluvastatin, can enhance sorafenib induced cytotoxicity in melanoma cells [34], [35]. Given, however, that sorafenib is not void of toxicities, and given the availability of more effective RAF and VEGF-R2 inhibitors, careful consideration should be given to clinical investigation of sorafenib-based combinations and thorough pre-clinical studies are warranted prior to proceeding to clinical trials.

Predictive biomarkers have not been previously studied for the carboplatin and paclitaxel regimen in melanoma. Our results suggest that the candidate biomarkers found here should be further validated on additional patients treated with this regimen. In addition, we note that given the design of this study, we are unable to differentiate between predictive versus prognostic biomarkers in this cohort, as there was no arm that did not receive treatment. Of the biomarkers studied, the only one with known predictive value for any systemic therapy is B-Raf mutations in patients treated with inhibitors of mutant B-Raf [6]. The presence of B-Raf mutations is also associated with poor prognosis in metastatic melanoma [36], [37].

In conclusion, an association was found between FGF-R1 and VEGF-R1 expression and OS, PFS and response to carboplatin/paclitaxel based therapy. Since sorafenib does not improve OS, PFS or response rate when added to carboplatin and paclitaxel, and seeing that carboplatin and paclitaxel use for melanoma has increased in recent years, particularly for B-Raf wild-type melanomas and patients who are not candidates for immune therapy, these biomarkers should be validated in additional cohorts, and their role as predictive versus prognostic biomarkers should be clarified in untreated patients. Moreover, more active and selective MAP kinase pathway inhibitors have recently emerged along with other promising drugs such as ipilimumab, and priority should be given to investigating such novel drugs or combinations thereof in patients with metastatic melanoma and to identifying biomarkers predictive of response to regimens with greater anti-tumor activity than SCP.

## Supporting Information

File S1 **Supporting Tables S1–S9 and Figures S1 and S2.**
(DOC)
