# Supplementary material for: Expression of Drug Targets in Patients Treated with Sorafenib, Carboplatin and Paclitaxel
Source: PLoS One. 2013 Aug 6;8(8):e69748. doi: 10.1371/journal.pone.0069748 (PMC3735539; doi:10.1371/journal.pone.0069748)
Supplement: File S1 — Supporting Tables S1–S9 and Figures S1 and S2. (DOC) [file pone.0069748.s001.doc]

**Table S1. List of Antibodies.**

| **Target Name** | **Source** | **Concentration** |
| --- | --- | --- |
| **MEK1** | **Upstate (rabbit monoclonal) 04-376** | **1:200** |
| **B-Raf** | **Santa Cruz (rabbit polyclonal) C-19** | **1:100** |
| **C-Raf** | **Upstate (rabbit monoclonal) 04-412** | **1:100** |
| **ERK1/2** | **Cell Signaling (mouse monoclonal) L34F12** | **1:400** |
| **VEGF-R1** | **Santa Cruz (rabbit polyclonal) C-17** | **1:200** |
| **VEGF-R2** | **Santa Cruz (mouse monoclonal) A-3** | **1:200** |
| **VEGF-R3** | **Santa Cruz (rabbit polyclonal) C-20** | **1:500** |
| **FGF-R1** | **QED Bioscience (mouse monoclonal) M5G10** | **1:100** |
| **PDGF-Rβ** | **BD Transduction (mouse monoclonal) C-28** | **1:30** |
| **CD117, c-Kit** | **Dako (rabbit polyclonal) A4502** | **1:50** |
| **STMN1** | **Epitomics (rabbit monoclonal) EP1573Y** | **1:1200** |
| **MAP2** | **Cell Signaling (rabbit polyclonal)** | **1:150** |
| **EB1** | **Cell Signaling (mouse monoclonal) 1A11/4** | **1:500** |
| **Bcl-2** | **Dako (mouse monoclonal) 124** | **1:30** |

**Table S2: Patient demographics and disease characteristics**

| Variable | Patients excluded (n=560) | | Patients included (n=263) | | P value | CP arm (140/263) | | SCP arm (123/263) | | P value |
| --- | --- | --- | --- | --- | --- | --- | --- | --- | --- | --- |
|  | n | % | n | % |  | n | % | n | % |  |
| Age (mean, SD) | 58.9 | 12.6 | 58.4 | 13.1 | 0.573 | 57.7 | 12.8 | 59.1 | 13.5 | 0.410 |
| Gender |  |  |  |  | 0.140 |  |  |  |  | 0.079 |
| Male | 345 | 61.6 | 176 | 66.9 |  | 87 | 62.1 | 89 | 72.4 |  |
| Female | 215 | 38.4 | 87 | 33.1 |  | 53 | 37.9 | 34 | 27.6 |  |
| Race |  |  |  |  | 0.660 |  |  |  |  | 0.887 |
| White | 544 | 97.1 | 254 | 96.6 |  | 135 | 96.4 | 119 | 96.8 |  |
| Other | 16 | 2.9 | 9 | 3.4 |  | 5 | 3.6 | 4 | 3.3 |  |
| AJCC stage |  |  |  |  | 0.428 |  |  |  |  | 0.945 |
| Unresectable Stage III | 44 | 7.9 | 27 | 10.3 |  | 14 | 10.0 | 13 | 10.6 |  |
| M1a/M1b | 189 | 33.8 | 92 | 35.0 |  | 48 | 34.3 | 44 | 35.8 |  |
| M1c | 327 | 58.4 | 144 | 54.8 |  | 78 | 55.7 | 66 | 53.7 |  |
| ECOG performance status |  |  |  |  | 0.343 |  |  |  |  | 0.561 |
| 0 | 341 | 61.0 | 173 | 65.8 |  | 92 | 65.7 | 81 | 65.9 |  |
| 1 | 218 | 39.0 | 90 | 34.2 |  | 48 | 34.3 | 42 | 34.2 |  |
| Prior therapy |  |  |  |  | 0.053 |  |  |  |  | 0.190 |
| None | 334 | 59.6 | 145 | 55.1 |  | 70 | 50.0 | 75 | 61.0 |  |
| IFN/IL-2/GM-CSF | 199 | 35.5 | 112 | 42.6 |  | 66 | 47.1 | 46 | 37.4 |  |
| One investigational therapy | 27 | 4.8 | 6 | 2.3 |  | 4 | 2.9 | 2 | 1.6 |  |
| Number of organs involved |  |  |  |  | 0.715 |  |  |  |  | 0.776 |
| 1 | 114 | 20.4 | 60 | 22.9 |  | 32 | 23.0 | 28 | 22.8 |  |
| 2-3 | 311 | 55.6 | 141 | 53.8 |  | 77 | 55.4 | 64 | 52.0 |  |
| >=4 | 134 | 24.0 | 61 | 23.3 |  | 30 | 21.6 | 31 | 25.2 |  |
| LDH |  |  |  |  | 0.291 |  |  |  |  | 0.278 |
| Normal | 303 | 56.7 | 156 | 60.7 |  | 88 | 63.8 | 68 | 57.1 |  |
| Above normal | 231 | 43.3 | 101 | 39.3 |  | 50 | 36.2 | 51 | 42.9 |  |
| Status of primary tumor |  |  |  |  | 0.965 |  |  |  |  | 0.935 |
| Unresected | 50 | 9.0 | 24 | 9.2 |  | 14 | 10.1 | 10 | 8.3 |  |
| Recurrent | 124 | 22.4 | 62 | 23.9 |  | 34 | 24.5 | 28 | 23.1 |  |
| Not recurrent, resected | 253 | 45.8 | 120 | 46.2 |  | 62 | 44.6 | 58 | 47.9 |  |
| Not recurrent, resected, residual | 33 | 6.0 | 13 | 5.0 |  | 6 | 4.3 | 7 | 5.8 |  |
| Primary tumor is unknown | 93 | 16.8 | 41 | 15.8 |  | 23 | 16.6 | 18 | 14.9 |  |

AJCC: The American Joint Committee on Cancer, ECOG: Eastern Cooperative Oncology Group, LDH: Lactate dehydrogenase, SCP: sorafenib, carboplatin and paclitaxel, CP: carboplatin and paclitaxel.

**Table S3: Outcomes in patients included in the biomarker study versus those who were excluded.**

| Outcomes | Patients excluded (560) | Patients included (263) | P value | CP arm (140/263) | SCP arm (123/263) | P value |
| --- | --- | --- | --- | --- | --- | --- |
| ORR (%, 95% CI) | 20.4 (17.1-23.9) | 17.1 (12.8-22.2) | 0.298 | 16.4 (10.7-23.6) | 17.9 (11.6-25.8) | 0.870 |
| PFS (median, 95% CI, months) | 4.7 (4.2-5.3) | 4.5 (3.4-5.4) | 0.692 | 3.7 (3.0-5.5) | 5.0 (3.8-5.8) | 0.525 |
| OS (median, 95% CI, months) | 11.5 (10.5-12.4) | 10.4 (9.2-11.6) | 0.960 | 11.4 (10.0-14.3) | 10.1 (8.5-11.0) | 0.154 |

Note: P values were generated by the Fisher exact test for ORR, and by the log rank test for PFS and OS. ORR: objective response rate, PFS: progression-free survival, OS: overall survival, CI: confidence interval SCP: sorafenib, carboplatin and paclitaxel, CP: carboplatin and paclitaxel.

**Table S4. Sample size and range of AQUA scores for each biomarker**

| **Marker** | **N** | **Responders** | **Non-Responders** | **Mean** | **Median** | **Min.** | **Max.** |
| --- | --- | --- | --- | --- | --- | --- | --- |
| **B-Raf** | 218 | 34 | 184 | 45.7 | 44.5 | 10.2 | 89.4 |
| **c-Kit** | 236 | 39 | 197 | 38.7 | 37.4 | 5.8 | 76.3 |
| **C-Raf** | 235 | 37 | 198 | 25.6 | 23.1 | 6.7 | 66.8 |
| **FGF-R1** | 254 | 42 | 212 | 68.9 | 68.3 | 28.8 | 127.8 |
| **VEGF-R1** | 225 | 36 | 189 | 34.2 | 31.7 | 5.5 | 88.1 |
| **VEGF-R3** | 239 | 39 | 200 | 52.1 | 50.2 | 12.9 | 143.9 |
| **MEK1** | 221 | 34 | 187 | 40.2 | 38.6 | 12.1 | 96.9 |
| **PDGF-Rβ** | 225 | 36 | 189 | 42.5 | 41.9 | 8.9 | 83.0 |
| **STMN1** | 197 | 27 | 170 | 55.8 | 55.5 | 11.8 | 110.9 |
| **MAP2** | 201 | 27 | 174 | 25.4 | 17.7 | 4.0 | 100.8 |
| **EB1** | 233 | 36 | 197 | 37.4 | 34.2 | 6.2 | 110.4 |
| **Bcl-2** | 233 | 37 | 196 | 26.4 | 20.3 | 3.2 | 88.4 |
| **ERK1/2** | 220 | 35 | 185 | 35.8 | 34.0 | 5.8 | 101.9 |
| **VEGF-R2** | 241 | 38 | 203 | 30.4 | 31.1 | 3.7 | 56.4 |

**Table S5. Association between biomarkers - Pearson correlation (R^2^ values)**

| **Markers** | **B-Raf** | **c-Kit** | **C-Raf** | **FGF-R1** | **VEGF-R1** | **VEGF-R3** | **MEK1** | **PDGF-Rβ** | **ERK1/2** | **VEGF-R2** |
| --- | --- | --- | --- | --- | --- | --- | --- | --- | --- | --- |
| **B-Raf** | 1 | 0.4325 | 0.5579 | 0.2323 | 0.4382 | 0.4729 | 0.4782 | 0.6405 | 0.4846 | 0.6263 |
| **c-Kit** | 0.4325 | 1 | 0.5571 | 0.574 | 0.5409 | 0.5502 | 0.4782 | 0.6018 | 0.4623 | 0.583 |
| **C-Raf** | 0.5579 | 0.5571 | 1 | 0.2447 | 0.3301 | 0.3831 | 0.5177 | 0.5791 | 0.5006 | 0.675 |
| **FGF-R1** | 0.2323 | 0.574 | 0.2447 | 1 | 0.5676 | 0.4796 | 0.2598 | 0.4615 | 0.2796 | 0.3911 |
| **VEGF-R1** | 0.4382 | 0.5409 | 0.3301 | 0.5676 | 1 | 0.5375 | 0.3042 | 0.532 | 0.4182 | 0.3878 |
| **VEGF-R3** | 0.4729 | 0.5502 | 0.3831 | 0.4796 | 0.5375 | 1 | 0.4609 | 0.5262 | 0.2038 | 0.4794 |
| **MEK1** | 0.4782 | 0.4782 | 0.5177 | 0.2598 | 0.3042 | 0.4609 | 1 | 0.5882 | 0.3064 | 0.4495 |
| **PDGF-Rβ** | 0.6405 | 0.6018 | 0.5791 | 0.4615 | 0.532 | 0.5262 | 0.5882 | 1 | 0.4268 | 0.6183 |
| **ERK1/2** | 0.4846 | 0.4623 | 0.5006 | 0.2796 | 0.4182 | 0.2038 | 0.3064 | 0.4268 | 1 | 0.3994 |
| **VEGF-R2** | 0.6263 | 0.583 | 0.675 | 0.3911 | 0.3878 | 0.4794 | 0.4495 | 0.6183 | 0.3994 | 1 |

**Table S6. Multivariable Cox regression model for association between OS and FGF-R1**

| **Variables** | **Level** | **HR** | **SE** | **z** | **P value** | **95%CI** |
| --- | --- | --- | --- | --- | --- | --- |
| **FGF-R1** | **High vs. Low** | **0.64** | **0.09** | **-3.11** | **0.0019** | **0.48-0.85** |
| AJCC stage | Stage III (ref.) |  |  |  |  |  |
|  | M1a/M1b | 0.86 | 0.22 | -0.60 | 0.5466 | 0.52-1.41 |
|  | M1c | 0.89 | 0.23 | -0.47 | 0.6394 | 0.54-1.47 |
| ECOG PS | 1 vs. 0 | 1.32 | 0.20 | 1.87 | 0.0613 | 0.99-1.77 |
| **Prior Treatment** | None (ref.) |  |  |  |  |  |
|  | IFN/IL-2/GM-CSF | 0.83 | 0.12 | -1.27 | 0.2042 | 0.62-1.11 |
|  | **One investigational therapy** | **2.31** | **0.98** | **1.96** | **0.0499** | **1.00-5.32** |
| Site number | 1 |  |  |  |  |  |
|  | 2-3 | 1.37 | 0.26 | 1.65 | 0.0991 | 0.94-2.00 |
|  | >=4 | 1.20 | 0.29 | 0.75 | 0.4543 | 0.75-1.92 |
| **LDH** | **Elevated vs. Normal** | **1.78** | **0.28** | **3.64** | **0.0003** | **1.31-2.43** |

HR: hazard ratio, SE: standard error, CI: confidence interval, AJCC: The American Joint Committee on Cancer, ECOG PS: Eastern Cooperative Oncology Group performance status, LDH: Lactate dehydrogenase.

**Table S7. Multivariable Cox regression model for association between OS and VEGF-R1**

| **Variables** | **Level** | **HR** | **SE** | **z** | **P value** | **95%CI** |
| --- | --- | --- | --- | --- | --- | --- |
| **VEGF-R1** | **High vs. Low** | **0.65** | **0.10** | **-2.76** | **0.0058** | **0.48-0.88** |
| AJCC stage | Stage III (ref.) |  |  |  |  |  |
|  | M1a/M1b | 0.74 | 0.19 | -1.15 | 0.2486 | 0.45-1.23 |
|  | M1c | 0.79 | 0.21 | -0.90 | 0.3662 | 0.47-1.32 |
| ECOG PS | 1 vs. 0 | 1.26 | 0.20 | 1.47 | 0.1418 | 0.92-1.73 |
| Prior treatment | None(ref.) |  |  |  |  |  |
|  | IFN/IL-2/GM-CSF | 0.80 | 0.12 | -1.45 | 0.1466 | 0.59-1.08 |
|  | One investigational therapy | 3.27 | 2.00 | 1.94 | 0.0524 | 0.99-10.85 |
| **Site number** | 1 |  |  |  |  |  |
|  | **2-3** | **1.51** | **0.30** | **2.07** | **0.0388** | **1.02-2.23** |
|  | >=4 | 1.40 | 0.36 | 1.31 | 0.1902 | 0.852.33 |
| **LDH** | **Elevated vs. Normal** | **1.64** | **0.28** | **2.95** | **0.0031** | **1.18-2.29** |

HR: hazard ratio, SE: standard error, CI: confidence interval, AJCC: The American Joint Committee on Cancer, ECOG PS: Eastern Cooperative Oncology Group performance status, LDH: Lactate dehydrogenase.

**Table S8. Multivariable Cox regression model for association between OS and c-Kit**

| **Variables** | **Level** | **HR** | **SE** | **z** | **P value** | **95%CI** |
| --- | --- | --- | --- | --- | --- | --- |
| **c-Kit** | **High vs. Low** | **0.71** | **0.10** | **-2.32** | **0.0205** | **0.53-0.95** |
| AJCC stage | Stage III (ref.) |  |  |  |  |  |
|  | M1a/M1b | 0.86 | 0.21 | -0.61 | 0.5386 | 0.52-1.40 |
|  | M1c | 0.79 | 0.20 | -0.94 | 0.3482 | 0.48-1.30 |
| ECOG PS | 1 vs. 0 | 1.32 | 0.21 | 1.79 | 0.0741 | 0.97-1.79 |
| Prior treatment | None(ref.) |  |  |  |  |  |
|  | IFN/IL-2/GM-CSF | 0.83 | 0.13 | -1.25 | 0.2115 | 0.61-1.11 |
|  | One investigational therapy | 2.06 | 0.96 | 1.55 | 0.1215 | 0.83-5.12 |
| **Site number** | 1 |  |  |  |  |  |
|  | **2-3** | **1.53** | **0.30** | **2.18** | **0.0290** | **1.04-2.25** |
|  | >=4 | 1.49 | 0.37 | 1.60 | 0.1091 | 0.92-2.42 |
| **LDH** | **Elevated vs. Normal** | **1.69** | **0.27** | **3.28** | **0.0010** | **1.23-2.31** |

HR: hazard ratio, SE: standard error, CI: confidence interval, AJCC: The American Joint Committee on Cancer, ECOG PS: Eastern Cooperative Oncology Group performance status, LDH: Lactate dehydrogenase.

**Table S9. Multivariable Cox regression model for association between PFS and FGF-R1**

| **Variables** | **Level** | **HR** | **SE** | **z** | **P value** | **95%CI** |
| --- | --- | --- | --- | --- | --- | --- |
| **FGF-R1** | **High vs. Low** | **0.71** | **0.10** | **-2.40** | **0.0165** | **0.54-0.94** |
| AJCC stage | Stage III (ref.) |  |  |  |  |  |
|  | M1a/M1b | 1.03 | 0.25 | 0.13 | 0.8957 | 0.64-1.67 |
|  | M1c | 1.23 | 0.30 | 0.84 | 0.4026 | 0.76-1.99 |
| ECOG PS | 1 vs. 0 | 1.17 | 0.17 | 1.08 | 0.2796 | 0.88-1.56 |
| Prior treatment | None(ref.) |  |  |  |  |  |
|  | IFN/IL-2/GM-CSF | 1.26 | 0.18 | 1.65 | 0.0987 | 0.96-1.66 |
|  | One investigational therapy | 2.35 | 1.04 | 1.93 | 0.0537 | 0.99-5.58 |
| Site number | 1 |  |  |  |  |  |
|  | 2-3 | 1.16 | 0.21 | 0.86 | 0.3918 | 0.82-1.65 |
|  | >=4 | 1.21 | 0.28 | 0.86 | 0.3925 | 0.78-1.90 |
| LDH | Elevated vs. Normal | 1.27 | 0.19 | 1.57 | 0.1165 | 0.94-1.71 |

HR: hazard ratio, SE: standard error, CI: confidence interval, AJCC: The American Joint Committee on Cancer, ECOG PS: Eastern Cooperative Oncology Group performance status, LDH: Lactate dehydrogenase.

**
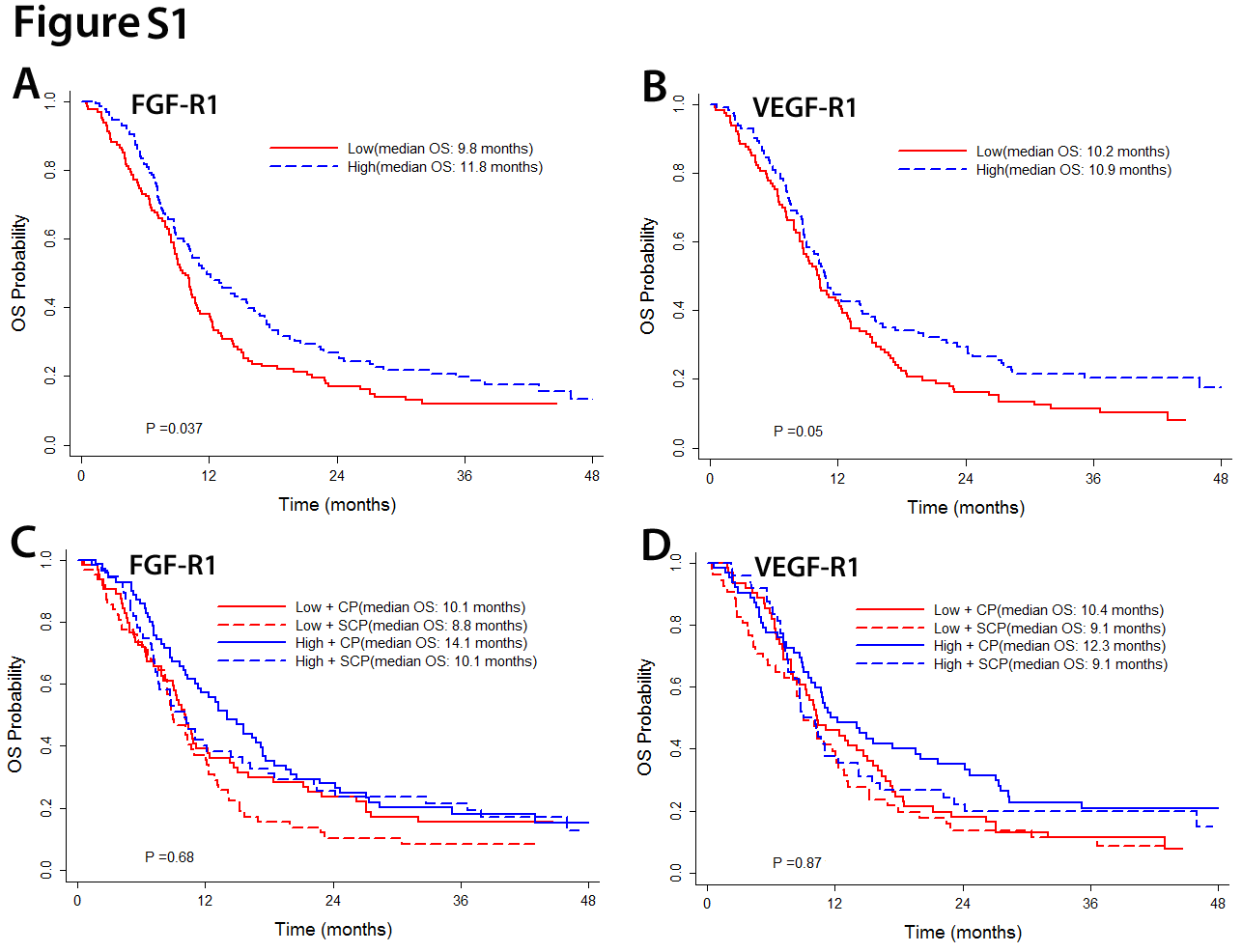
**

Kaplan-Meier survival curves for FGFR1 and VEGF-R1. Panels A and B show Kaplan-Meier estimates of OS by dichotomized FGF-R1 and VEGF-R1, respectively (p values were generated from log rank tests). Panels C and D show Kaplan-Meier estimates of OS by treatment and dichotomized FGF-R1 and VEGF-R, respectively (p values were generated from treatment-by-maker interaction tests in Cox models). SCP: sorafenib, carboplatin and paclitaxel, CP: carboplatin and paclitaxel.

**
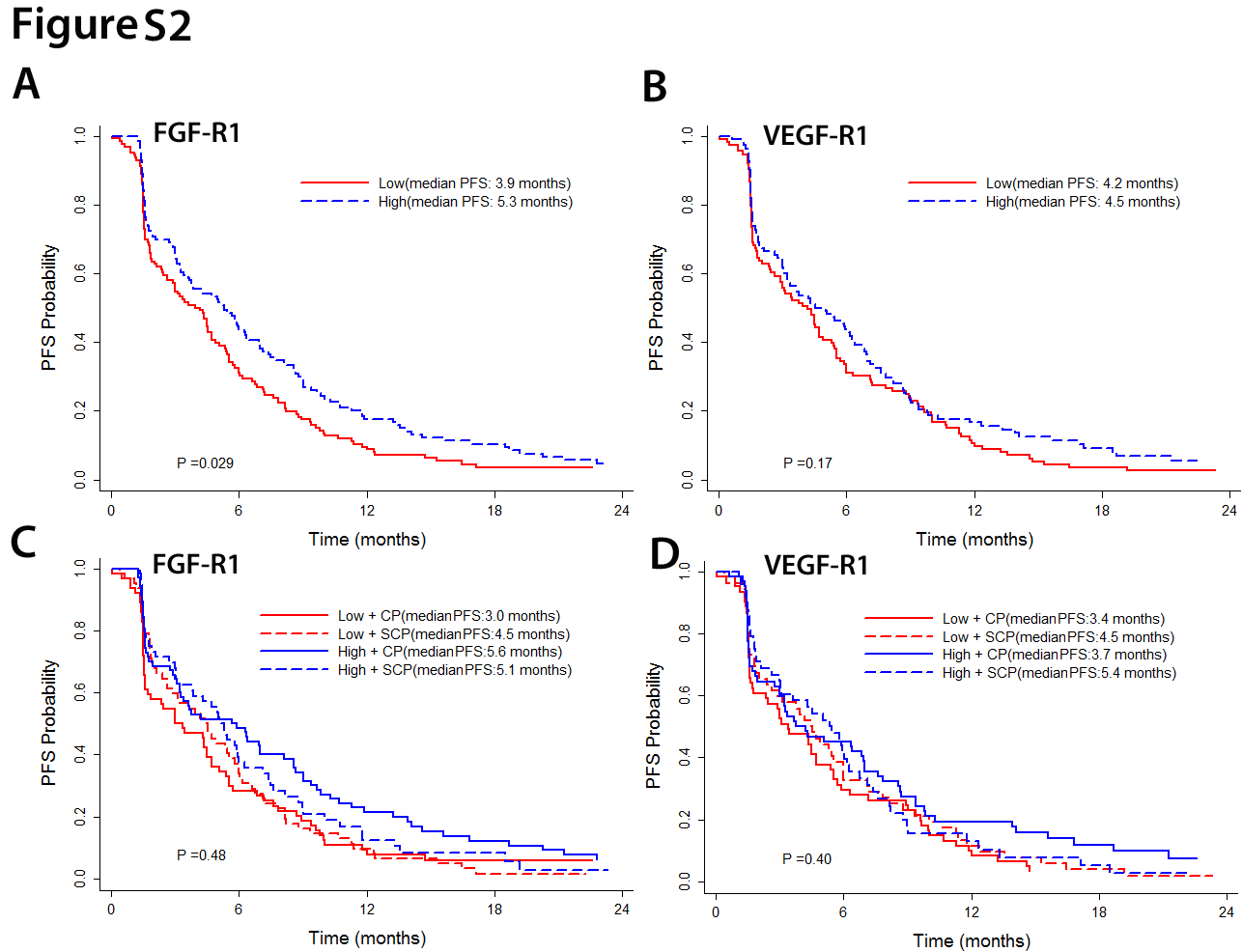
**

Kaplan-Meier survival curves for FGFR1 and VEGF-R1. Panels A and B show Kaplan-Meier estimates of PFS by dichotomized FGF-R1 and VEGF-R1, respectively (p values were generated from log rank test). Panels C and D show Kaplan-Meier estimates of PFS by treatment and dichotomized FGF-R1 and VEGF-R, respectively (p values were generated from treatment-by-maker interaction test in Cox models). SCP: sorafenib, carboplatin and paclitaxel, CP: carboplatin and paclitaxel.
